# Corneal Posterior Curvature Changes After Phacoemulsification Cataract Surgery with 2.75 mm Corneal Incision

**Published:** 2019

**Authors:** Artur Jose SCHMITT, Ana Tereza Ramos MOREIRA, Faruk Abrão Kalil FILHO, Fernanda Piccoli SCHMITT

**Affiliations:** 1 Ophthalmology Department, Universidade Federal do Paraná (UFPR), Curitiba, PR, Brazil; 2 Hospital Barigui de Oftalmologia, Curitiba, PR, Brazil

**Keywords:** Phacoemulsification, Corneal Incision, Corneal Tomography, Corneal Posterior Curvature

## Abstract

The aim of this study was to evaluate the corneal posterior curvature changes after phacoemulsification cataract surgery, with intraocular lens implantation, with a temporal limbal self-sealing 2.75 millimeters (mm) corneal incision, using a Placido-dual rotating Scheimpflug device. In this prospective intervention study, corneal posterior curvature changes were evaluated in fifty-six patients (56 eyes). All patients underwent corneal tomography using the Galilei G2 (Ziemer Ophthalmic System AG, Port, Switzerland) preoperatively (PRE) and with two weeks (RP15), one month (RP30), and three months (RP90) after phacoemulsification cataract surgery with a temporal limbal self-sealing 2.75 mm incision. Tomographic parameters analyzed in the posterior cornea were the steep curvature (K2), flat curvature (K1), mean curvature (average K), and posterior corneal astigmatism. We did not observe any statistically significant change in the K2, K1, average K, and posterior corneal astigmatism in any postoperative follow-up measurements (RP15, RP30, RP90), showing that the postoperative values tend to be the same as the preoperative ones when measured with the Galilei G2 tomography. In conclusion, the 2.75 mm temporal limbal self-sealing corneal incision in phacoemulsification cataract surgery does not induce significant changes in the posterior corneal curvature parameters of K2, K1, average K, and astigmatism.

## INTRODUCTION

As the refractive surface of the cornea is responsible for the major optical power of the eye, its surface has a crucial role in the optical function of the human visual system. This performance is outlined by the shape, regularity, clarity and refractive index of the cornea [1]. Therefore, changes in these elements can change the visual acuity. 

Cataract is the most prevalent cause of reversible blindness worldwide. The only curative treatment of cataracts is surgical intervention and consists of replacing the cloudy lens by an intraocular lens (IOL) [2]. Cataract surgery may be performed by various techniques including phacoemulsification (PHACO) and extracapsular extraction [3]. The PHACO is the most used technique in cataract surgery in the world. It has the advantages of the smaller incision, less trauma to the eye, as well as shorter operation time and visual recovery [3]. Corneal astigmatism occurs frequently after cataract surgery, with 15% to 50% of patients with cataract having 1- 2 diopter (D) corneal astigmatism following surgery [4, 5]. Surgical advances in the latest years with the presentation of toric IOLs have allowed surgical correction of astigmatism after cataract surgery, resulting in better results regarding the quality of uncorrected vision, yet a considerable number of patients still present with higher than predicted residual cylinder values [6, 7]. Though several aspects are implied in post-surgical astigmatism [6, 7], one of the factors studied newly is related to the estimation methods of total corneal astigmatism and its relation to the posterior cornea [6, 7]. Therefore, recent investigations have been done to address the effect of posterior corneal astigmatism on total corneal astigmatism, and its influence on post-surgical astigmatism [6, 7]. 

Our study intends to evaluate the corneal posterior curvature changes after phacoemulsification cataract surgery, with IOL implantation, with a temporal limbal self-sealing 2.75 millimeters (mm) corneal incision, using a Placido-dual rotating Scheimpflug device.

## METHODS

This was a prospective intervention study performed under the coordination of the Department of Ophthalmology, Federal University of Parana, on Curitiba, Brazil. The research design of this study was approved by the Ethics Committee on Human Research of the Health Sciences Sector of the Federal University of Paraná with the number 1990476. From June 2016 to December 2016, individuals diagnosed with cataracts who underwent surgical treatment for cataract surgery using PHACO were invited to participate in this study. The inclusion criteria were individuals diagnosed with cataracts who underwent surgical treatment for cataract surgery by PHACO and absence of situations that presented prior corneal changes (rigid contact lenses users, refractive surgery, corneal trauma, corneal transplantation, keratoconus, and corneal ulcer). Exclusion criteria were need to use suture on the cornea after the procedure, vitreous loss during cataract surgery with or without the need for an anterior vitrectomy procedure or pars plana vitrectomy, unable to attend the appointments of the postoperative period and need for reoperation. 

All patients were informed about the diagnosis of cataract, the need for surgical treatment and its relation to the improvement of visual acuity. The patients were also informed about the examination of corneal tomography. Patients who agreed to participate signed the informed consent form and underwent a complete ophthalmic examination consisting of refraction, visual acuity, intraocular pressure, the ocular surface and the anterior segment assessment using slit lamp and examination of the retina under mydriasis. In addition to the full ophthalmic examination, all patients were submitted to additional preoperative tests including corneal topography, specular microscopy exam, optical biometry and corneal tomography (Galilei dual Scheimpflug system G2, Ziemer Ophthalmic System AG, Port, Switzerland). All examinations were performed by the same technique and analyzed by the same observer.

**Surgical Technique:** Patients enrolled in the study underwent surgery in the Ophthalmology Barigui Hospital with all operations performed by the same surgeon (AJS), using a surgical microscope and the same surgical procedure in all patients. Pupillary dilation was performed with instillation of tropicamide 1% every 15 minutes, starting 60 minutes before initiation of operation. After anesthesia with peribulbar block, and installation of aseptic surgical drapes, Barraquer blepharostat was placed. The procedures for cataract surgery started with the surgeon sitting laterally (temporal position) in relation to the operated eye. A corneal incision of 2.75 mm was made at 8 o'clock in the right eye and 2 o'clock in the left eye. Paracentesis of 1 mm was made at 10 o'clock in the right eyes and 4 o'clock in the left eyes. The incision and paracentesis were located at 0.5 mm anterior to a corneal limbus. The incision was performed using a 2.75 mm disposable scalpel.

The paracentesis was performed using a 1mm disposable scalpel. A continuous curvilinear capsulorhexis measuring approximately 5.0 mm in diameter was created after the anterior chamber was filled with viscoelastic substance. After hydrodissection of the lens, the nucleus was removed using phacoemulsification equipment (*Centurion, Alcon Laboratories, Fort Worth, The USA*). The cortex was removed using the automated aspiration system from the phacoemulsification Centurion. After this aspiration, the capsular bag was filled with the same viscoelastic substance used previously and the IOL (*C Flex 570 C, Rayner, Hove, England)* implantation was performed through the 2.75 mm incision. After removal of the viscoelastic, paracentesis and incision were sealed with standard hydration with the balanced salt solution. Then instillation of antibiotic eye drops and corticosteroids (gatifloxacin 3 mg/mL, and prednisolone acetate 10 mg/mL) were performed. Postoperatively the patients were oriented to use drops of Gatifloxacin 3 mg/mL, prednisolone acetate 10 mg/mL 4 times per day for 15 days and ketorolac trometamol drops 0.5% 3 times a day for 30 days. After surgical procedures, patients were followed routinely with ophthalmic examinations on the first day, 15, 30 and 90 days after surgery. Also, corneal tomography was performed on the fifteen (postoperative [RP15]), thirty (RP30) and ninety (RP90) days postoperatively.

The analysis of corneal tomography was based on realization of an axial map of the posterior corneal curvature using the *Galilei G2*. The parameters evaluated were the posterior curvature of the cornea in the flattest meridian (K1), the posterior corneal curvature of the steepest meridian (K2), the average posterior curvature of the cornea (average K) and posterior corneal astigmatism.

Results of Quantitative variables were described as means and standard deviations (SD). For statistical analysis, we used the Past Program (Paleontological Statistics Version 3.25, University of Oslo, Norway). To test the normality of data, the Shapiro-Wilk W test was used with significance level of 5% (normal distribution P >0.05). For values with normal distribution Student's t-Test was used with a significance level of 5% (P < 0.05). For values with non-normal distribution Mann Whitney test with a significance level of 5% (P < 0.05) was used. For astigmatism values, we used Student t-test and the Mann Whitney test for mean values of average K, K1 and K2. We tested the null hypothesis that the means of all variables (astigmatism, average K, K1 and K2) are equal in the four moments of evaluation; preoperative (PRE), RP15, RP30 and RP90 against the alternative hypothesis that the least one of the four moments has different mean preoperatively.

## RESULTS

There were 60 patients diagnosed with cataract having surgical treatment indication. One eye of each patient was evaluated consisting a total of 60 eyes. Of this total, we excluded four patients due to non-attendance at the correct time of the consultations to perform postoperative corneal tomography. In total, 56 patients completed all the stages of the study. Of whom, 26 (46.4%) were female and 30 (53.6%) male. Regarding the affected eye, the right eye was affected in 39 patients (69.6%) and the left eye in 17 patients (30.4%). The mean± SD of the age of patients undergoing surgery was 67.2 ± 12.6 years old, the youngest patient was 31 years old and the oldest was 87 years old. 

Regarding the K2, the mean± SD in the PRE period was -6.47 Diopter (D) 0.36 D, on the RP15 was -6.52 D 0.33 D, on the RP30 was -6.48 D 0.34 D and on the RP90 was -6.48 D 0.34 D. There was no statistically significant variation in K2 value comparing K2 preoperatively value with the postoperative values (RP15, RP30, and RP90). When comparing K2 preoperatively with K2 on the RP15, P value was 0.36, comparing K2 preoperatively with K2 on the RP30, P value was 0.88 and comparing K2 preoperatively with K2 on the RP90, P value was 0.96 (Table 1)

Regarding the K1, the mean± SD in the PRE period was -6.12 D 0.35 D, on the RP15 was -6.20 D 0.34 D, on the RP30 was -6.18 D 0.32 D and on the RP90 was -6.13 D 0.33 D. There was no statistically significant variation in K1 value comparing K1 preoperatively value with the postoperative values (RP15, RP30 and RP90). When comparing K1 preoperatively with K2 on the RP15, P value was 0.37 comparing K1 preoperatively with K1 on the RP30, P value was 0.59, when comparing K1 preoperatively with K1 on the RP90, P value was 0.89 (Table 2).

Regarding the average K, the mean± SD in the PRE period was -6.30 D 0.33 D, on the RP15 was -6.35 D 0.34 D, on the RP30 was -6.33 D 0.31 D and on the RP90 was -6.30 D 0.32 D. There was no statistically significant change in the average K value comparing average K preoperatively with the postoperative values (RP15, RP30, and RP90). When comparing average K preoperatively with the average K on the RP15, P value was 0.37, comparing average K preoperatively with average K on RP30, P value was 0.59 and comparing average K preoperatively with the average K on the RP90, P value was 0.37 (Table 3).

The value of PRE total astigmatism was -0.34 D 0.14 D, on the RP15 was -0.32 D 0.14 D, on the RP30 was -0.31 D 0.13 D and at RP90 was -0.35 D 0.15 D. There was no statistically significant variation in the values of the posterior corneal astigmatism comparing PRE astigmatism with the other times (RP15, RP30, and RP90). When comparing astigmatism of the PRE time with the astigmatism on the RP15, P value was 0.50, comparing astigmatism of the PRE time with astigmatism on RP30 P value was 0.29, comparing astigmatism of the PRE time with astigmatism on the RP90, P value was 0.50(Table 4).

**Table 1:** Comparison of Pre and Postoperative K2 Values

	RP15 VS PRE	RP30 VS PRE	RP90 VS PRE
Mann Whitney (P)	0.36	0.88	0.96
Significant difference	Not	Not	Not
PRE (D) Mean± SD	-6.47 ± 0.36		
RP (D) Mean± SD	-6.52 ± 0.33	-6.48 ± 0.34	-6.48 ± 0.34

K2: the steepest meridian; RP15: fifteen days postoperative; RP30: thirty days postoperative; RP90: ninety days postoperative; PRE: preoperative; RP: postoperative; VS: versus; SD: standard deviation; D: diopter; P: P value. P < 0.05 considered significant.

**Table 2:** Comparison of Pre and Postoperative K1 Values

	RP15 VS PRE	RP30 VS PRE	RP90 VS PRE
Mann Whitney (P)	0.37	0.59	0.89
Significant difference	Not	Not	Not
PRE (D) Mean± SD	-6.12 ± 0.35		
RP (D) Mean± SD	-6.20 ± 0.34	-6.18 ± 0.32	-6.13 ± 0.33

K1: the flattest meridian; RP15: fifteen days postoperative; RP30: thirty days postoperative; RP90: ninety days postoperative; PRE: preoperative; RP: postoperative; VS: versus; SD: standard deviation; D: diopter; P: P value. P < 0.05 considered significant.

**Table 3:** Comparison of Pre and Postoperative Average K

	RP15 VS PRE	RP30 VS PRE	RP90 VS PRE
Mann Whitney (p)	0.37	0.59	0.37
Significant difference	Not	Not	Not
PRE (D) Mean ± SD	-6.30 ± 0.33		
RP (D) Mean ± SD	-6.35 ± 0.34	-6.33 ± 0.31	-6.30 ± 0.32

RP15: fifteen days postoperative; RP30: thirty days postoperative; RP90: ninety days postoperative; PRE: preoperative; RP: postoperative; VS: versus; SD: standard deviation; D: diopter; P: P value. P < 0.05 considered significant.

**Table 4:** Comparison of Pre and Postoperative Astigmatism

	RP15 VS PRE	RP30 VS PRE	RP90 VS PRE
t-test (P)	0.50	0.29	0.50
Significant difference	Not	Not	Not
PRE (D) Mean ± SD	-0.34 ± 0.14		
RP (D) Mean ± SD	-0.32 ± 0.14	-0.31 ± 0.13	-0.35 ± 0.15

RP15: fifteen days postoperative; RP30: thirty days postoperative; RP90: ninety days postoperative; PRE: preoperative; RP: postoperative; VS: versus; SD: standard deviation; D: diopter; P: p-value. P<0.05 considered significant.

## DISCUSSION

Our study assessed whether there is a significant change on parameters of posterior corneal following phacoemulsification cataract surgery with a 2.75 mm temporal cornea incision. We did not observe any statistically significant change in the K2, K1, average K and the posterior corneal astigmatism in any postoperative follow-up measurements (RP15, RP30, RP90), suggesting that the postoperative values tend to be the same as the preoperative ones when measured with the Galilei G2 tomography. 

Like any surgical procedure, certain complications are possible. The major complications related to cataract surgeries are opacification of the posterior capsule, decentration, anterior or posterior dislocation, pupillary membrane and IOL phimosis, inflammation, elevated intraocular pressure, posterior capsule rupture with or without vitreous loss, cystoid macular edema, posterior synechiae, incarceration of the iris and opacification of the IOL [8]. In this study, there were no postoperative complications. Publications demonstrated that cataract surgery may alter the biomechanical properties of the cornea and this change can be related to the size of surgical incisions [9]. Corneal incisions generally modify previous corneal astigmatism. This change is dependent on the size, shape and location of the incision [10]. The change of the corneal curvature is less when applying scleral incision, temporal location and a cut length less than 2 mm [11]. Only 2% of cataract surgeries are performed with micro incisions (< 2 mm), which are neutral for astigmatism, 66% of cataract surgeries are performed with incisions between 2.6 to 3.1 mm [12]. In our research, we used 2.75 mm incisions. The 2.75 mm incision generates a change of 0.65 D in the anterior curvature [13]. However, the impact of corneal incisions in the posterior curvature of the cornea is still unclear [14].

Until recently, surgeons have not considered relevant the posterior corneal astigmatism because precise measurement of the corneal posterior surface has not been possible. Another reason was the small difference of refractive indices between the posterior corneal surface to the aqueous humor. With the advancement of the types of equipment that measure the posterior corneal curvature, there is a growing interest in the knowledge of changes of the posterior curvature of the cornea following cataract surgery [15]. In the present study, we found no published scientific studies concerning changes of the posterior cornea with corneal incision of 2.75 mm. The posterior corneal curvature is clinically relevant and often underestimated in clinical practice for correction of astigmatism. Studies reported that not considering the posterior curvature of the cornea results in refractive errors postoperatively [15, 16].

Refractive errors after cataract surgery with results not expected with toric IOLs are among the most dissatisfaction factors for ophthalmic surgeons. These negative results may be partially explained when neglected the individual effect of posterior astigmatism and its subsequent change in eye surgery [15]. In our study, we observed mean posterior astigmatism of -0.34 D. This value is similar to that found by other authors [17, 18], using different methods of analysis of the corneal curvature (Purkinje and Scheimpflug images) finding posterior corneal astigmatism of -0.26 D to -0.78. The value found in our research is also close to the value of - 0.30 D found by another research when using the same measurement equipment of our research (Galilei) for analyzing the posterior curvature [16]. We did not find a statistically significant variation in values of the preoperative posterior astigmatism compared to other moments (RP15, RP30, and RP90) measured postoperatively in the present study. The non-significant variation of the posterior corneal astigmatism using 2.75 mm corneal incision in cataract surgery, has an important significance in the calculation of IOLs, especially IOLs toric. Posterior astigmatism contributes more significantly to ocular astigmatism we imagine and has implications on how we correct the astigmatism of patients with relaxing incisions or, more importantly, with toric IOLs [16].

Correction of astigmatism with toric IOLs has become a standard practice for many eye surgeons, but visual results are not always predicted accurately. One source of uncertainty in the surgical process is the posterior corneal astigmatism. Between 70% and 87% of eyes have with the rule astigmatism in the posterior curvature of -0.3 to -0.5 D. As a result, in most patients the posterior cornea increases the total amount of ocular against the rule astigmatism, because the posterior corneal curvature is negative, justifying the need for evaluation when planning to implant toric IOLs [16]. In our study, because no variation in posterior astigmatism after corneal incision of 2.75 mm was observed, it can be assumed that the preoperative posterior curvature values can be considered in the surgical planning of IOLs Toric without a change correction factor by the incision. Regarding the results of this study in which no level of significance of the variables was observed, the final values after surgery (RP90) were similar to initial values of preoperative in relation to the values of the posterior corneal curvature both in the variation of values K1, K2, total astigmatism and mean K compared to the other postoperative values (PRE, RP15, RP30, and RP90).

All data tended to return to the initial values of the research, so no change was observed on the posterior curvature of the cornea after surgery with phacoemulsification incision of 2.75 mm. 

Some hypotheses can be raised to justify our result. Collagen in the corneal surface and its viscoelasticity that is responsible for any aggression that may generate an elastic deformation is reversible by cessation of stress, returning to its original shape and volume [18] More keratocytes presented on the front surface than the back one can also justify the difference in results regarding variation of anterior and posterior corneal curvature after corneal incision [15]. Another hypothesis is the anatomy of the cornea. The Descemet's membrane is a basement membrane of endothelial cells. It can be easily separated from the stroma. Once this structure is traumatized, the edges recede indicating an inherent elasticity. Histologically, it is a homogeneous structure with glass aspect, ultrastructurally is comprised of stratified layers of very fine filaments of collagen. This layer is capable of rapid regeneration [19], hence values of posterior curvature return to preoperative ones after corneal incisions of 2.75mm. The difference of the anatomy and physiology of the cornea on the posterior face compared to its anterior one may be related to the study results, showing that its posterior structure can be returned to its initial morphological state after 90 days of surgery the same as preoperative values using this size incision surgery. 

Despite the novelity of current research which investigated changes of posterior corneal curvature with corneal incision of 2.75 mm, our limitations were population size being relatively small (56 eyes) and short term follow-up (90 days). Further investigations with more patients are necessary to establish more comprehensive results. Also, a long term follow-up can help to understand delayed changes in posterior corneal curvature.

## CONCLUSIONS

We conclude that there was no significant change in the posterior cornea curvature when 2.75 mm corneal incision was used in phacoemulsification cataract surgery, on 15, 30 and 90 days after surgery.
